# Factors influencing the optimal glycemic levels in type 2 diabetes mellitus patients attending the diabetic clinic of a tertiary care hospital in New Delhi

**DOI:** 10.1186/s12902-026-02417-5

**Published:** 2026-07-10

**Authors:** Ishant Kumar, Sanjeet Panesar, Rishi Kumar Jaiswal, Neelam Roy, Deepak Dhamnetiya

**Affiliations:** 1 Department of Community Medicine, School of Medical Sciences and Research, Sharda University, Knowledge Park III, Greater Noida, Gautam Buddha Nagar, Uttar Pradesh 201310 India; 2 Atal Bihari Vajpayee Institute of Medical Sciences and Dr. Ram Manohar Lohia Hospital, Baba Kharak Singh Marg, Connaught Place, New Delhi, 110001 India

**Keywords:** Type 2 diabetes mellitus, Glycaemic control, Medication adherence, Lifestyle factors, HbA1c

## Abstract

**Background:**

Optimal glycaemic control is essential for preventing complications of type 2 diabetes mellitus (T2DM), yet a large proportion of patients fail to achieve recommended glycaemic targets even in tertiary care settings. Identifying independent behavioural and treatment-related determinants of glycaemic control is crucial for improving diabetes management.

**Methods:**

A hospital-based analytical cross-sectional study was conducted among 416 adult patients with T2DM attending the diabetic outpatient department of a tertiary care hospital. Data were collected using a structured, pre-tested questionnaire covering sociodemographic profile, lifestyle practices, treatment adherence, and healthcare access. Glycaemic control was assessed using HbA1c values and categorized as optimal (< 7%) and poor (≥ 7%). Descriptive statistics, chi-square test, Spearman correlation, and multivariable logistic regression were applied using IBM SPSS Statistics for Mac (Version 31.0).

**Results:**

Optimal glycaemic control was observed in 239 (57.5%) participants, while 177 (42.5%) had poor control. Higher physical activity, adherence to a diabetic diet, and consistent medication adherence were significantly associated with optimal glycaemic control in bivariate analysis (*p* < 0.05). Spearman correlation showed a strong positive correlation between medication adherence and glycaemic control, followed by physical activity and regular follow-up. In multivariable regression analysis, medication adherence, physical activity, and regular follow-up emerged as significant independent predictors of optimal glycaemic control, whereas sociodemographic factors showed weaker adjusted effects.

**Conclusion:**

Glycaemic control among patients with T2DM is predominantly influenced by modifiable behavioural and treatment-related factors. Strengthening medication adherence counselling, lifestyle modification, and structured follow-up within tertiary care diabetes clinics may substantially improve glycaemic outcomes and reduce the long-term burden of diabetes-related complications.

**Clinical trial number:**

Not applicable.

**Supplementary Information:**

The online version contains supplementary material available at https://doi.org/10.1186/s12902-026-02417-5.

## Introduction

Diabetes mellitus has emerged as one of the most significant non-communicable diseases globally, posing a major public health challenge due to its rising prevalence, chronic complications, and substantial economic burden. According to the International Diabetes Federation, the global prevalence of diabetes among adults has increased steadily over the past decades, with low- and middle-income countries, including India, contributing a major share of the disease burden [1]. India, often referred to as the “diabetes capital of the world,” is witnessing a rapid epidemiological transition characterized by increasing urbanization, sedentary lifestyles, and dietary shifts, all of which contribute to the rising incidence of type 2 diabetes mellitus (T2DM) [2].

Optimal glycaemic control is a cornerstone in the management of T2DM, as sustained hyperglycaemia is strongly associated with microvascular and macrovascular complications such as retinopathy, nephropathy, neuropathy, cardiovascular diseases, and increased mortality [3]. Glycated hemoglobin (HbA1c) is widely recognized as the gold standard indicator for long-term glycaemic control, reflecting average blood glucose levels over the preceding two to three months [4]. Clinical guidelines generally recommend maintaining HbA1c levels below 7% to reduce the risk of diabetes-related complications and improve quality of life [5].

Despite the availability of effective pharmacological therapies and structured diabetes care programs, achieving optimal glycaemic control remains a challenge in real-world settings, particularly in developing countries [6]. Multiple factors influence glycaemic outcomes, including sociodemographic characteristics, lifestyle behaviours, treatment adherence, access to healthcare, and psychosocial determinants [7]. Evidence suggests that age, gender, socioeconomic status, and place of residence can significantly affect diabetes self-management and glycaemic control [8].

Lifestyle-related factors such as physical inactivity, unhealthy dietary patterns, smoking, and poor adherence to diabetic diets have been consistently associated with poor glycaemic control [9]. Regular physical activity improves insulin sensitivity and glycaemic regulation, while adherence to a structured diabetic diet plays a crucial role in maintaining metabolic balance [10]. Conversely, sedentary behaviour and non-adherence to dietary recommendations are linked with higher HbA1c levels and disease progression [11].

Treatment-related factors also play a pivotal role in determining glycaemic outcomes. Medication adherence, regular follow-up visits, and timely modification of treatment regimens are essential components of effective diabetes management [12]. Studies have shown that inconsistent medication adherence and irregular clinic attendance are major contributors to suboptimal glycaemic control among patients with T2DM [13]. Furthermore, barriers to healthcare access, including financial constraints, lack of awareness, and healthcare system limitations, may hinder optimal disease management [14].

In tertiary care settings, where patients often present with long-standing diabetes and multiple comorbidities, understanding the interplay between lifestyle practices, treatment adherence, and healthcare utilization becomes particularly important [15]. However, there is limited context-specific evidence from tertiary care hospitals in India examining the combined influence of sociodemographic, behavioural, and treatment-related factors on glycaemic control.

Although previous studies have examined isolated determinants of glycaemic control, most have focused on either demographic characteristics, lifestyle factors, or treatment adherence independently. Limited evidence is available from Indian tertiary care settings evaluating the combined influence of sociodemographic, behavioural, lifestyle, and treatment-related factors on glycaemic outcomes within a single analytical framework. Furthermore, studies integrating bivariate, correlation, and multivariable analyses to identify independent predictors of glycaemic control in routine clinical settings remain scarce. Addressing this evidence gap is important for developing targeted interventions and strengthening diabetes management strategies in tertiary care practice.

Therefore, the present study was conducted to assess the glycaemic control status and its association with selected sociodemographic characteristics, lifestyle factors, and treatment-related determinants among patients with type 2 diabetes mellitus attending a tertiary care diabetic clinic. The findings of this study are expected to provide evidence for targeted interventions aimed at improving diabetes self-management and optimizing glycaemic outcomes in clinical practice.

## Methodology

### Study design and setting

This hospital-based analytical cross-sectional study was conducted over a period of eight months, from July 2024 to February 2025 to assess the factors influencing optimal glycaemic control among patients with type 2 diabetes mellitus attending the diabetic clinic of a tertiary care teaching hospital in New Delhi, India. The study was implemented in the Diabetic Outpatient Department (OPD), which caters to a large and diverse patient population from urban, peri-urban, and rural catchment areas. The cross-sectional design enabled the simultaneous assessment of glycaemic control status and its association with sociodemographic, lifestyle, and treatment-related determinants at a single point in time. The quantitative approach facilitated the identification of measurable relationships between behavioural practices, treatment adherence, and glycaemic outcomes, thereby providing clinically relevant evidence for improving diabetes management in tertiary care settings.

### Study duration

The study was conducted over a defined data collection period of eight months, following approval from the Institutional Ethics Committee and administrative permission from the hospital authorities. Data collection was carried out on all working clinic days to ensure adequate participant coverage and representation of routine diabetic clinic attendees.

### Study area

The present study was conducted at the Diabetic Clinic of Dr. Ram Manohar Lohia Hospital, New Delhi, a tertiary care government teaching hospital that serves a high patient load from the National Capital Region (NCR) and adjoining states. The hospital provides specialized diabetes care, including routine glycaemic monitoring, pharmacological management, lifestyle counselling, and regular follow-up services.

New Delhi, located in the northern part of India, is a major metropolitan region characterized by rapid urbanization, mixed socioeconomic profiles, and a high burden of non-communicable diseases, particularly diabetes mellitus. The patient population attending the diabetic clinic comprises individuals from diverse occupational, educational, and socioeconomic backgrounds, including salaried employees, retirees, homemakers, and informal sector workers. The availability of structured diabetes care services, laboratory facilities for HbA1c testing, and standardized treatment protocols made the setting appropriate for examining determinants of glycaemic control in a real-world clinical context.

### Participants

The study population comprised adult patients diagnosed with type 2 diabetes mellitus who attended the diabetic OPD during the study period. Participants were enrolled based on predefined inclusion and exclusion criteria to ensure uniformity and validity of responses.

Inclusion criteria were: (a) patients aged 18 years and above, (b) clinically diagnosed cases of type 2 diabetes mellitus, (c) those who provided informed consent for participation. Exclusion criteria included: (a) patients with type 1 diabetes mellitus or gestational diabetes, (b) critically ill patients unable to participate in interviews, (c) individuals with severe cognitive impairment or psychiatric illness affecting comprehension, and (d) patients who declined consent.

### Sampling and data collection

A consecutive sampling technique was employed. All eligible patients attending the diabetic clinic during the study period and fulfilling the inclusion criteria were approached for participation until the desired sample size was achieved. This method ensured feasibility in a high patient-load tertiary care setting while maintaining adequate representation of routine clinic attendees. In cases where a patient refused participation or did not meet eligibility criteria, the next eligible patient was recruited to maintain continuity of sampling.

The sample size was calculated using the standard formula for prevalence studies, assuming a 95% confidence level, an absolute precision of 4.5%, and an expected prevalence of optimal glycaemic control of approximately 31% among patients with type 2 diabetes mellitus [16], which reported that nearly one-third of diabetic individuals achieved HbA1c < 7%. Substituting these values yielded a minimum required sample size of approximately 406 participants. Considering the hospital-based design, possible non-response, and the need to ensure adequate statistical power for subgroup and association analyses, a total of 416 patients were included in the final analysis. The achieved sample size thus met and slightly exceeded the calculated requirement, enhancing the precision and reliability of the study estimates.

Data were collected through structured face-to-face interviews conducted in a private setting within the outpatient department to ensure confidentiality and reduce response bias. A pretested and semi-structured questionnaire was used for data collection. The tool was administered using EpiCollect (Version 5), which facilitated real-time data entry, validation, and secure storage.

Participants were first briefed about the objectives, voluntary nature, and confidentiality of the study. Verbal informed consent was obtained prior to the interview. Each interview lasted approximately 15–20 min. Efforts were made to minimize recall bias by cross-verifying treatment and HbA1c records from patient medical files wherever available.

To ensure methodological consistency, all interviews were conducted using standardized procedures by trained investigators following predefined interview protocols. Data collection procedures were pilot tested prior to study implementation to improve reproducibility and reduce interviewer-related variability.

Potential information bias was minimized through the use of structured interviews and cross-verification of HbA1c values and treatment details from available medical records whenever feasible. Interviews were conducted in a private setting to reduce social desirability bias.

### Study variables and operational definitions

The primary outcome variable was glycaemic control, assessed using the most recent HbA1c value recorded in the patient’s medical record. Glycaemic control was categorized as optimal (HbA1c < 7%) and poor (HbA1c ≥ 7%) in accordance with standard clinical guidelines.

Sociodemographic variables included age, gender, place of residence (urban/rural), family type (nuclear/joint), and socioeconomic status. Socioeconomic status was classified using a standardized socioeconomic scale appropriate for urban Indian settings. Lifestyle-related variables included physical activity (frequency per week), adherence to a diabetic diet (yes/no), and smoking status (current smoker/non-smoker). Physical activity was categorized based on self-reported weekly frequency. Treatment-related variables included pharmacological treatment status, medication adherence (very consistent, somewhat consistent, not consistent), and regularity of follow-up visits to the diabetic clinic (regular, occasional, rare). Barriers to healthcare access were also assessed as a dichotomous variable based on self-reported difficulties in accessing services.

### Measurement tool

Data were collected using a structured, pre-tested questionnaire developed specifically for this study and provided as Supplementary File 1. The questionnaire comprised four sections: sociodemographic characteristics, lifestyle factors, medication and treatment adherence, and healthcare access and support. The tool was designed to capture key behavioural and clinical determinants influencing glycaemic control among patients with type 2 diabetes mellitus.

The sociodemographic section included items on age, gender, religion, residence (urban/rural), family type (nuclear/joint), educational level, occupation, income, and duration of diabetes diagnosis. These variables were used to assess the background characteristics and social determinants potentially associated with glycaemic outcomes. Lifestyle factors were assessed using self-reported items on physical activity frequency per week, adherence to a specific diabetic dietary plan, daily consumption of fruits and vegetables, home blood glucose monitoring practices, and smoking status. Physical activity was categorized into four levels (less than once a week, 1–2 times/week, 3–4 times/week, and ≥ 5 times/week) to reflect varying intensity of lifestyle engagement. Dietary adherence and smoking were recorded as dichotomous variables (yes/no), while glucose monitoring frequency was categorized based on routine practice. Medication and treatment adherence were evaluated using items on current pharmacological treatment status, consistency in taking prescribed medication (very consistent, somewhat consistent, not consistent), missed doses, and challenges in accessing medications or treatment. These items were used to operationalize treatment adherence as a key predictor variable.

The healthcare access and support section included items on frequency of follow-up visits to the diabetic clinic (regular, occasional, rare), satisfaction with healthcare team support, and perceived barriers to accessing diabetes-related healthcare services. All responses were coded into categorical variables for statistical analysis. The questionnaire was administered through face-to-face interviews to ensure clarity and completeness of responses. Content validity was established through expert review by faculty members with expertise in Community Medicine and diabetes care. The questionnaire was pilot tested among a subset of participants prior to final implementation to assess clarity, contextual suitability, and feasibility. Necessary modifications were incorporated before final data collection. The complete English version of the questionnaire has been provided as Supplementary File 1 to facilitate transparency and reproducibility.

### Descriptive and inferential statistics

Normality assessment of continuous variables was performed using graphical methods and distribution characteristics prior to inferential analysis. Statistical assumptions for regression analysis were evaluated before model development. Data were entered, coded, and cleaned using Microsoft Excel, followed by statistical analysis using IBM SPSS Statistics. Descriptive statistics such as frequencies, percentages, means, and standard deviations were calculated to summarize sociodemographic characteristics, lifestyle factors, treatment adherence, and glycaemic control status. Continuous variables were presented as mean ± standard deviation, while categorical variables were expressed as frequencies and percentages.

### Bivariate analysis

The association between glycaemic control and selected sociodemographic, lifestyle, and treatment-related variables was examined using the Chi-square test. Cross-tabulations were performed to compare the proportion of participants with optimal and poor glycaemic control across different categories such as gender, socioeconomic status, physical activity, dietary adherence, smoking status, and medication adherence. A p-value of less than 0.05 was considered statistically significant.

### Multivariable analysis

Multicollinearity among independent variables was assessed prior to model estimation and found to be within acceptable limits. Variables included in the regression model were selected based on theoretical relevance, previous literature, and statistically significant bivariate associations. Regression modelling was used to control for potential confounding effects and identify independent predictors of optimal glycaemic control.

### Software

Data entry and preliminary data cleaning were performed using Microsoft^®^ Excel for Mac, Version 16.93.1 (25011917; Microsoft Corporation, Redmond, WA, USA). All statistical analyses were conducted using IBM SPSS Statistics for Mac, Version 31.0.0.0 (117) (IBM Corp., Armonk, NY, USA). Publication-quality tables and graphical visualizations were prepared using SPSS and Python-based plotting tools and exported in high-resolution formats suitable for journal submission.

### Ethical considerations

The study received ethical approval from the Institutional Ethics Committee of ABVIMS, Dr. RML Hospital, New Delhi (File. No. 724(38/2024)/IEC/ABVIMS/RMLH)/419, the Institutional Ethics Committee prior to commencement. Verbal informed consent was obtained from all participants after explaining the purpose, procedures, potential benefits, and voluntary nature of participation in their preferred language. Participants were assured of confidentiality and anonymity, and no personally identifiable information was recorded. Each questionnaire was assigned a unique identification code, and all data were stored in password-protected files accessible only to the research team. The study was conducted in accordance with the ethical principles outlined in the Declaration of Helsinki.

## Results

A total of 416 patients with type 2 diabetes mellitus were included, of whom 239 (57.5%) had optimal glycaemic control and 177 (42.5%) had poor control. The mean age was 51.8 ± 14.8 years. Males constituted 232 (55.8%) of participants. Most participants were urban residents (312; 75.0%) and belonged to nuclear families (264; 63.5%). The upper-middle socioeconomic class predominated (240; 57.7%) (Table 1).

**Table 1:** Sociodemographic characteristics of study participants (*n* = 416)

Variable	Frequency (%) / Mean ± SD
Age (years)	51.8 ± 14.8
Male	232 (55.8)
Female	176 (42.3)
Urban residence	312 (75.0)
Rural residence	104 (25.0)
Nuclear family	264 (63.5)
Joint family	152 (36.5)
Upper class	80 (19.2)
Upper middle class	240 (57.7)
Lower middle class	72 (17.3)
Upper lower class	24 (5.8)

Physical activity was high in a substantial proportion of participants, with 160 (38.5%) reporting activity five or more times per week and 96 (23.1%) reporting activity three to four times per week, while 32 (7.7%) reported no physical activity. Adherence to a diabetic diet was reported by 216 participants (51.9%). Smoking was reported by 88 participants (21.2%). Most participants were receiving pharmacological treatment (392; 94.2%), with 328 (78.8%) reporting high medication adherence. Regular clinic follow-up was reported by 336 participants (80.8%), while barriers to healthcare access were reported by 264 (63.5%). (Table 2).

**Table 2:** Lifestyle, treatment, and healthcare characteristics of study participants (*n* = 416)

Variable	Frequency (%)
Physical activity ≥ 5 times/week	160 (38.5)
Physical activity 3–4 times/week	96 (23.1)
Physical activity 1–2 times/week	64 (15.4)
Physical activity < 1 time/week	64 (15.4)
No physical activity	32 (7.7)
Following diabetic diet – Yes	216 (51.9)
Following diabetic diet – No	200 (48.1)
Smokers	88 (21.2)
Non-smokers	328 (78.8)
On antidiabetic medication – Yes	392 (94.2)
On antidiabetic medication – No	24 (5.8)
Very consistent medication adherence	328 (78.8)
Somewhat consistent medication adherence	72 (17.3)
Not consistent medication adherence	16 (3.8)
Regular follow-up	336 (80.8)
Occasional follow-up	72 (17.3)
Rare follow-up	8 (1.9)
Barriers to healthcare access – Yes	264 (63.5)
Barriers to healthcare access – No	152 (36.5)

The association between glycaemic control and selected factors is shown in Table 3. Optimal glycaemic control was observed in 239 participants (57.5%). Male gender was significantly associated with better glycaemic control (χ² = 14.40, *p* = 0.001), as was higher socioeconomic status (χ² = 9.31, *p* = 0.025), while place of residence showed no significant association (χ² = 1.18, *p* = 0.277). Higher levels of physical activity and adherence to a diabetic diet were strongly associated with optimal glycaemic control (χ² = 59.01 and 13.34 respectively; both *p* < 0.001), whereas smoking status was not (χ² = 1.51, *p* = 0.219). Medication adherence demonstrated the strongest association, with consistently adherent participants showing markedly better glycaemic control (χ² = 107.45, *p* < 0.001).

**Table 3:** Association between selected factors and glycaemic control status (*n* = 416)

Variable	Optimal control *n* (%)	Poor control *n* (%)	Chi-square (χ²)	*p*-value
Gender	239 (57.5)	177 (42.5)	14.40	0.001
Residence	239 (57.5)	177 (42.5)	1.18	0.277
Socioeconomic scale	239 (57.5)	177 (42.5)	9.31	0.025
Physical activity	239 (57.5)	177 (42.5)	59.01	< 0.001
Dietary adherence	239 (57.5)	177 (42.5)	13.34	< 0.001
Smoking	239 (57.5)	177 (42.5)	1.51	0.219
Medication adherence	239 (57.5)	177 (42.5)	107.45	< 0.001

**Fig. 1:**
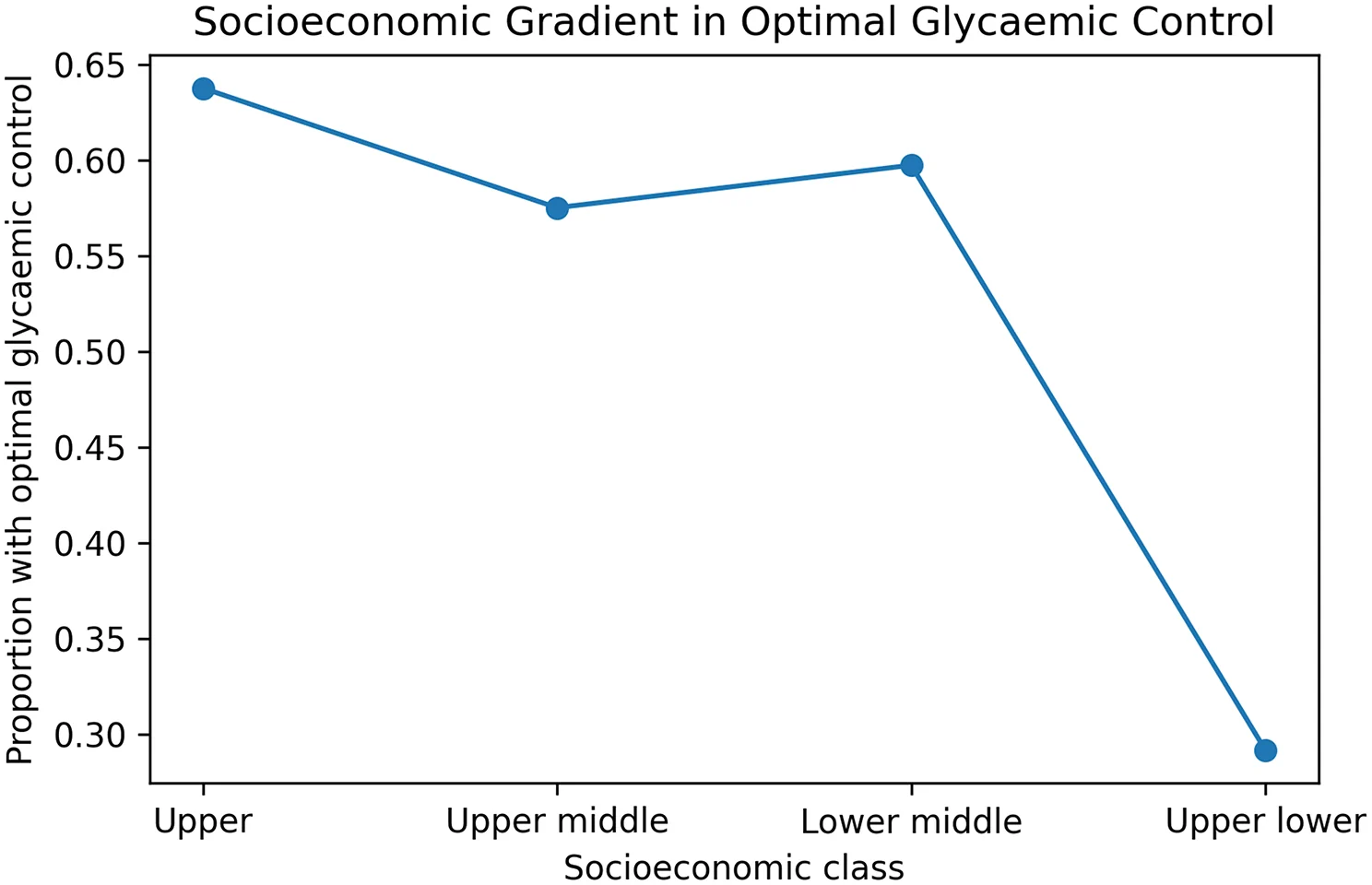
Socioeconomic gradient in glycaemic control

Figure 1 illustrates the distribution of optimal glycaemic control across socioeconomic categories. A clear socioeconomic gradient was observed, with a higher proportion of participants achieving optimal glycaemic control in the upper and upper-middle socioeconomic classes compared to lower socioeconomic categories. The proportion of optimal glycaemic control progressively declined among participants belonging to lower-middle and upper-lower socioeconomic groups, indicating poorer glycaemic outcomes with decreasing socioeconomic status.

The Table 4 shows, on multivariable logistic regression analysis, regular physical activity (≥ 3 times per week) was independently associated with optimal glycaemic control (adjusted odds ratio [aOR] 4.01; 95% CI: 2.53–6.37; *p* < 0.001). Male gender was also independently associated with optimal glycaemic control (aOR 1.70; 95% CI: 1.06–2.70; *p* = 0.027). Smoking was negatively associated with optimal glycaemic control (aOR 0.46; 95% CI: 0.26–0.81; *p* = 0.007). Age and dietary adherence were not significantly associated with glycaemic control after adjustment.

**Table 4:** Multivariable logistic regression analysis for optimal glycaemic control (HbA1c < 7%)

Variable	Adjusted OR (95% CI)	*p*-value
Age (years)	1.01 (0.99–1.03)	0.125
Male gender	1.70 (1.06–2.70)	0.027
Regular physical activity (≥ 3 times/week)	4.01 (2.53–6.37)	< 0.001
Dietary adherence	1.30 (0.83–2.04)	0.259
Smoking	0.46 (0.26–0.81)	0.007

In Table 5, Spearman correlation analysis showed a strong positive correlation between medication adherence and glycaemic control (*r* = 0.67, *p* < 0.001), followed by physical activity (*r* = 0.49, *p* < 0.001) and regular follow-up (*r* = 0.41, *p* < 0.001). Age demonstrated a moderate negative correlation with glycaemic control (*r* = − 0.31, *p* < 0.001). Socioeconomic status showed a weak but significant positive correlation with glycaemic control (*r* = 0.24, *p* < 0.001).

**Table 5:** Pairwise correlation between age, physical activity, medication adherence, follow-up regularity, socioeconomic status and glycaemic control

Variables	Age	Physical activity (ord)	Medication adherence (ord)	Follow-up regularity (ord)	SES (ord)	Glycaemic control (code)
Age	1.00	-0.28 (*p* < 0.001)	-0.22 (*p* < 0.001)	-0.19 (*p* = 0.002)	0.11 (*p* = 0.041)	-0.31 (*p* < 0.001)
Physical activity (ord)	-0.28 (*p* < 0.001)	1.00	0.46 (*p* < 0.001)	0.38 (*p* < 0.001)	0.21 (*p* < 0.001)	0.49 (*p* < 0.001)
Medication adherence (ord)	-0.22 (*p* < 0.001)	0.46 (*p* < 0.001)	1.00	0.52 (*p* < 0.001)	0.18 (*p* = 0.001)	0.67 (*p* < 0.001)
Follow-up regularity (ord)	-0.19 (*p* = 0.002)	0.38 (*p* < 0.001)	0.52 (*p* < 0.001)	1.00	0.16 (*p* = 0.004)	0.41 (*p* < 0.001)
SES (ord)	0.11 (*p* = 0.041)	0.21 (*p* < 0.001)	0.18 (*p* = 0.001)	0.16 (*p* = 0.004)	1.00	0.24 (*p* < 0.001)
Glycaemic control (code)	-0.31 (*p* < 0.001)	0.49 (*p* < 0.001)	0.67 (*p* < 0.001)	0.41 (*p* < 0.001)	0.24 (*p* < 0.001)	1.00

* Higher glycaemic control code = optimal control; Ord = ordinal coding for ranked variables

**Fig. 2:**
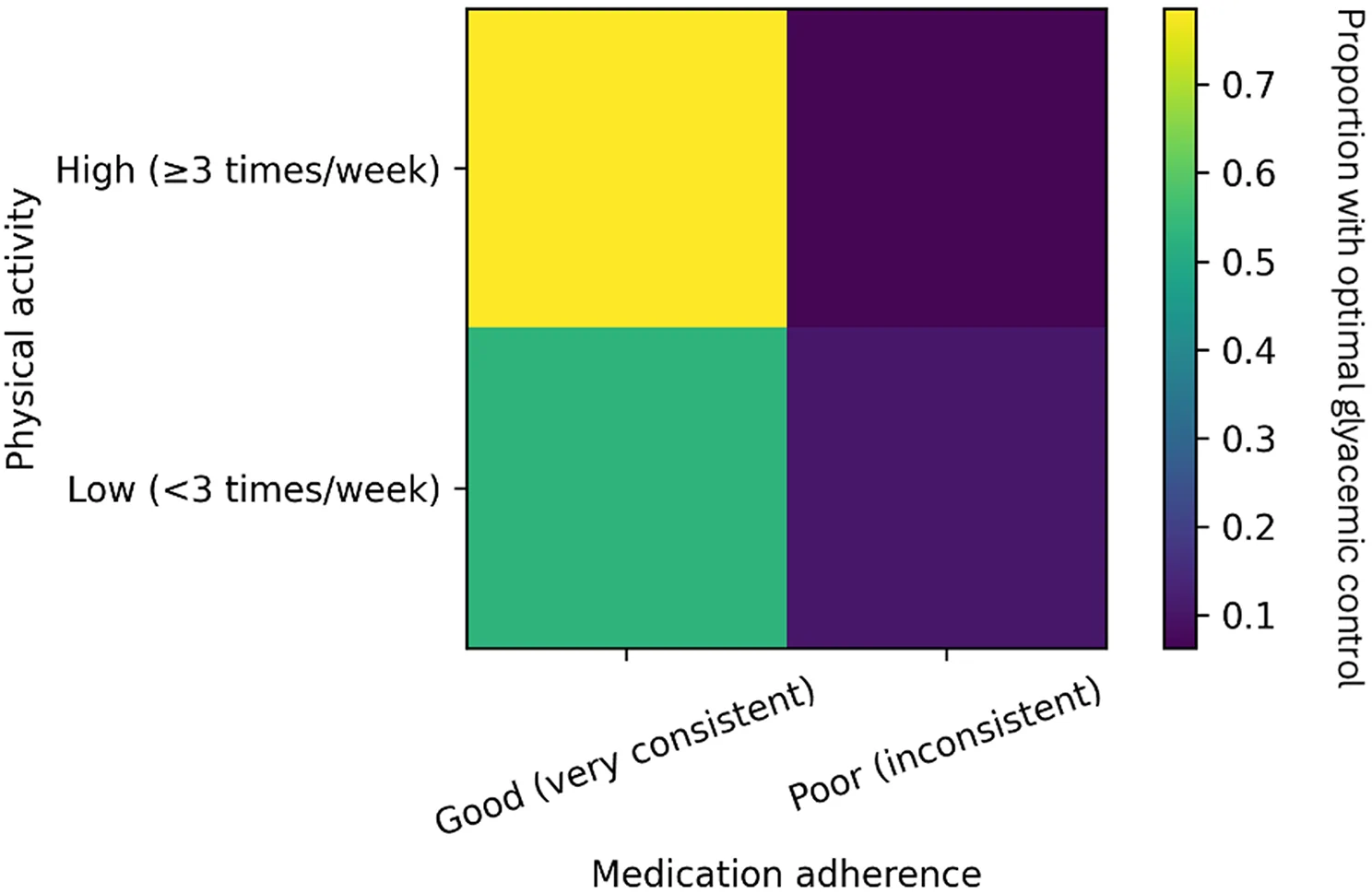
Combined effect of physical activity and medication adherence

Figure 2 depicts the combined influence of physical activity and medication adherence on glycaemic control. Participants reporting both regular physical activity and very consistent medication adherence demonstrated the highest proportion of optimal glycaemic control. In contrast, poor glycaemic control was most prevalent among participants with low physical activity levels and inconsistent medication adherence. Intermediate patterns were observed when either physical activity or medication adherence alone was favourable, highlighting the additive effect of lifestyle and treatment adherence on glycaemic outcomes.

## Discussion

The present study evaluated glycaemic control and its sociodemographic, lifestyle, and treatment-related determinants among patients with type 2 diabetes mellitus attending a tertiary care diabetic clinic. The findings showed that slightly more than half of the participants achieved optimal glycaemic control, while a substantial proportion remained poorly controlled, indicating persistent gaps in diabetes management even in a specialized care setting.

The study population largely comprised middle-aged individuals, which is consistent with the epidemiological pattern of type 2 diabetes in India and other developing countries where the disease predominantly affects individuals in their productive years [17]. The predominance of urban residents in the present study also reflects the growing urban burden of diabetes, likely attributable to sedentary lifestyles, dietary transitions, and occupational stress associated with urbanization [18]. Recent evidence from the ICMR-INDIAB-17 study indicates that India is experiencing a substantial increase in metabolic non-communicable diseases, with over 100 million individuals affected by diabetes and rapidly increasing prevalence across urban and rural settings. These findings underscore the importance of identifying modifiable determinants of glycaemic control among individuals with established diabetes [19].

Gender demonstrated a significant association with glycaemic control in the bivariate analysis, with males showing comparatively better control than females. Similar gender-based differences in diabetes self-management and health-seeking behaviour have been reported in previous studies [20]. However, existing literature also suggests that gender differences in glycaemic outcomes may vary across sociocultural contexts and healthcare access patterns, which may explain the variability observed across studies [21].

Socioeconomic status showed a significant gradient with glycaemic control, with participants from higher socioeconomic categories exhibiting better glycaemic outcomes. This finding is in line with earlier research indicating that individuals with higher socioeconomic status have better access to healthcare services, improved treatment affordability, and higher health literacy, all of which contribute to better diabetes management [22]. Conversely, lower socioeconomic status has consistently been associated with poorer glycaemic control due to financial constraints, limited healthcare accessibility, and reduced continuity of care [23]. However, in the multivariable regression analysis, the independent effect of socioeconomic status was attenuated, suggesting that its influence may be mediated through behavioural and adherence-related pathways.

Lifestyle-related factors demonstrated a strong relationship with glycaemic outcomes. Participants engaging in higher levels of physical activity and adhering to a diabetic diet were more likely to achieve optimal glycaemic control, and these associations remained clinically relevant after adjustment. These findings are consistent with established evidence showing that regular physical activity enhances insulin sensitivity and improves glycaemic regulation, while structured dietary adherence plays a central role in long-term metabolic control [24, 25]. In contrast, smoking did not show a significant independent association with glycaemic control in the present study, which is comparable to some previous studies reporting inconsistent relationships between smoking status and glycaemic outcomes in clinical populations [26].

Recent findings from ICMR-INDIAB-23 further indicate that metabolic dysfunction among Asian Indians may extend beyond conventional obesity definitions, with metabolically adverse phenotypes increasingly recognized among individuals without overt obesity. Such observations suggest that future studies should explore phenotype-based risk stratification approaches in diabetes management [27].

Treatment-related factors emerged as the most influential determinants of glycaemic control. Medication adherence demonstrated the strongest independent association with optimal glycaemic control in the regression analysis, highlighting the critical importance of pharmacological compliance in diabetes management. This observation is strongly supported by existing literature indicating that poor medication adherence is a major predictor of uncontrolled diabetes, increased hospitalization, and long-term complications [28]. Similarly, regular follow-up visits to the diabetic clinic were associated with better glycaemic outcomes, likely due to periodic monitoring, timely dose adjustments, and reinforcement of lifestyle counselling [29].

A considerable proportion of participants also reported barriers in accessing healthcare services, which may contribute to suboptimal glycaemic control despite availability of tertiary care facilities. Previous studies from low- and middle-income settings have highlighted those financial constraints, logistical difficulties, and limited awareness act as significant barriers to effective diabetes management and continuity of care [30]. These barriers may indirectly affect adherence, follow-up behaviour, and overall treatment outcomes.

Dietary behaviour and self-monitoring strategies play important roles in glycaemic optimization. Emerging evidence suggests that individualized dietary counselling and continuous glucose monitoring may facilitate improved treatment adherence and glycaemic outcomes, although such advanced monitoring approaches were beyond the scope of the present study.

Overall, the findings highlight the multifactorial nature of glycaemic control, wherein modifiable behavioural and treatment-related factors exert stronger independent influence than fixed sociodemographic characteristics. The integration of correlation analysis and multivariable regression strengthens the analytical robustness of the findings and provides evidence for identifying clinically relevant predictors of glycaemic outcomes.

### Limitations

This study has certain limitations that should be considered while interpreting the findings. First, the cross-sectional design limits causal inference despite the use of multivariable regression analysis. Second, the study was conducted in a single tertiary care hospital, which may restrict generalizability to primary care or community settings. Third, several key variables, including physical activity, dietary adherence, and medication consistency, were self-reported and may be subject to recall and social desirability bias. Despite multivariable adjustment, residual confounding from unmeasured variables cannot be entirely excluded. Finally, clinical parameters such as BMI, complications, and detailed treatment regimens were not included in the model, which may have further influenced glycaemic outcomes. Detailed pharmacological treatment characteristics, fixed-dose drug combinations, and advanced glucose monitoring methods such as continuous glucose monitoring were not evaluated and therefore their influence on glycaemic control could not be explored.

### Policy implications

The inclusion of multivariable regression analysis provides important policy-relevant insights by identifying independent predictors of glycaemic control. Since medication adherence and lifestyle practices emerged as key independent determinants, diabetes control programs should prioritize structured adherence counselling, behavioural interventions, and regular follow-up systems in tertiary care clinics. Integration of individualized lifestyle modification plans, digital adherence reminders, and continuous patient education under national NCD programs could significantly improve glycaemic outcomes. Additionally, strengthening diabetes counselling services and ensuring uninterrupted access to medications may yield greater impact than focusing solely on demographic risk profiling. Targeted interventions for physically inactive and non-adherent patients may substantially reduce the burden of poor glycaemic control in clinical populations.

## Conclusion

In conclusion, while more than half of the patients achieved optimal glycaemic control, a substantial proportion remained poorly controlled, indicating ongoing challenges in diabetes management. Multivariable regression analysis identified medication adherence, physical activity, and regular follow-up as key independent predictors of optimal glycaemic control, whereas sociodemographic factors showed comparatively weaker effects after adjustment. These findings highlight that modifiable behavioural and treatment-related factors play a dominant role in glycaemic outcomes. Strengthening adherence-focused counselling, lifestyle interventions, and structured follow-up mechanisms within tertiary care diabetes clinics may significantly improve long-term glycaemic control and reduce the risk of diabetes-related complications.

## Supplementary Information

Below is the link to the electronic supplementary material.

Supplementary Material 1

## Data Availability

The study datasets are coded and available from the corresponding author on reasonable request.

## Abbreviations

T2DM: Type 2 Diabetes Mellitus
HbA1c: Glycated Hemoglobin
OPD: Outpatient Department
WHO: World Health Organization
ADA: American Diabetes Association
NCDs: Non–Communicable Diseases
SPSS: Statistical Package for the Social Sciences
IDF: International Diabetes Federation
IEC: Information, Education and Communication
