# Supplementary material for: Factors influencing the optimal glycemic levels in type 2 diabetes mellitus patients attending the diabetic clinic of a tertiary care hospital in New Delhi
Source: BMC Endocr Disord. 2026 Jul 10;26:306. doi: 10.1186/s12902-026-02417-5 (PMC13644162; doi:10.1186/s12902-026-02417-5)
Supplement: Supplementary file 1 [file 12902_2026_2417_MOESM1_ESM.docx]

Supplementary File 1. Structured Questionnaire: **“Factors Influencing Optimal Glycaemic Levels Among Patients with Type 2 Diabetes Mellitus Attending a Tertiary Care Diabetic Clinic, New Delhi”**

Instructions to Participants: This questionnaire is intended to collect information regarding your lifestyle, treatment adherence, and healthcare access related to diabetes management. All responses will be kept confidential and used only for research purposes. Please answer all questions honestly. There are no right or wrong answers.

Section I: Identification Details
Unique Participant Code: __________

Sociodemographic Information

1. Age (in completed years): __________
2. Gender:
   Male
   Female
   Transgender
   Others (please specify): __________
3. Religion:
   Hindu
   Muslim
   Sikh
   Christian
   Others (please specify): __________
4. Marital Status:
   Single
   Married
   Widowed
   Separated
   Divorced
   Live-in relationship
5. Place of Residence:
   Urban
   Rural
6. Family Type / Living Arrangement:
   Nuclear family
   Joint family
   Living alone
7. Educational Level:
   Postgraduate/Professional degree
   Graduate
   Senior Secondary School
   Secondary School
   Middle School
   Primary School
   Illiterate
8. Occupation:
   Professional (e.g., doctor, engineer, senior administrative officer, advocate, business executive)
   Semi-professional (e.g., lecturer, insurance inspector)
   Clerical/Shop owner/Farmer
   Skilled worker (e.g., carpenter, mason, mechanic, driver, technician)
   Semi-skilled worker (e.g., factory labourer, assistant staff)
   Unskilled worker (e.g., manual labourer, peon, watchman)
   Unemployed/Homemaker
9. Monthly Family Income (INR) (as per socioeconomic classification):
   ≥ ₹249,044
   ₹124,489 – ₹249,043
   ₹93,381 – ₹124,488
   ₹62,273 – ₹93,380
   ₹37,325 – ₹62,272
   ₹12,445 – ₹37,324
   ≤ ₹12,444

Section II: Lifestyle Factors

1. How often do you engage in physical activity (e.g., exercise, brisk walking) per week?
   Never
   Less than once a week
   1–2 times per week
   3–4 times per week
   5 or more times per week
2. Do you follow a specific dietary plan for managing your diabetes?
   Yes
   No
   If yes, please specify: __________
3. On average, how many servings of fruits and vegetables do you consume daily?
   Less than 2 servings
   2–4 servings
   5 or more servings
   Do not consume regularly
4. How often do you monitor your blood glucose levels at home?
   Daily
   Few times a week
   Rarely
   Never
5. Do you currently smoke?
   Yes
   No
   If yes, average number of cigarette packs/boxes per week: __________

Section III: Medication and Treatment Adherence

1. Are you currently taking medication to manage your diabetes?
   Yes
   No
2. If yes, how consistent are you in taking your prescribed medication?
   Very consistent
   Somewhat consistent
   Not consistent
3. Have you ever missed doses of your diabetes medication?
   Yes
   No
   If yes, please specify reasons (if any): __________
4. Do you face any challenges in accessing your diabetes medication or treatment?
   Yes
   No
   If yes, please specify: __________
5. Latest HbA1c Level (%): __________

Section IV: Healthcare Access and Support

1. How often do you attend follow-up appointments at the diabetic clinic?
   Regularly
   Occasionally
   Rarely
2. Are you satisfied with the support and information provided by your healthcare team?
   Yes
   No
3. Do you face any barriers in accessing healthcare services for managing your diabetes?
   Yes
   No
   If yes, please specify the barriers: __________
